# Transcranial direct current stimulation of the right temporoparietal junction impairs third-person perspective taking

**DOI:** 10.3758/s13415-016-0462-z

**Published:** 2016-09-20

**Authors:** Michiel van Elk, Monique Duizer, Ilja Sligte, Hein van Schie

**Affiliations:** 1 Department of Psychology, University of Amsterdam, Nieuwe Achtergracht 129B, 1018WT Amsterdam, The Netherlands; 2 Behavioral Science Institute, Radboud University Nijmegen, Nijmegen, The Netherlands

**Keywords:** Spatial perspective taking, Mental body transformation task, Transcranial direct current stimulation, Right temporoparietal junction

## Abstract

Given the current debates about the precise functional role of the right temporoparietal junction (rTPJ) in egocentric and exocentric perspective taking, in the present study we manipulated activity in the rTPJ to investigate the effects on a spatial perspective-taking task. Participants engaged in a mental body transformation task, requiring them to mentally rotate their own body to the position of an avatar, while undergoing anodal, cathodal, or sham transcranial direct current stimulation (tDCS) of the rTPJ. As a control task, participants judged the laterality of a stimulus feature with respect to a fixation cross on the screen. For the first half of the experiment (only during online tDCS), a task-selective effect of tDCS was observed, reflected in slower reaction times following anodal than following cathodal and sham tDCS for the mental body transformation task, but not for the control task. The effects of tDCS were most pronounced for stimuli implying a more difficult mental body transformation. No effects of tDCS were observed during the second half of the experiment. The effects of tDCS were most pronounced for participants scoring low on aberrant perceptual beliefs and spiritual transcendence, suggesting a relation between third-person perspective taking and bodily and perceptual experiences. The finding that anodal stimulation of the rTPJ impairs third-person perspective taking indicates a key role of this region in exocentric spatial processing.

## Introduction

When interacting with others, we often need to distinguish between our own and others’ perspectives. For instance, when riding a bike one needs to infer what others can see, while at the same time maintaining a first-person or egocentric perspective to coordinate one’s own actions. It has been argued that spatial perspective taking and the ability to switch between first- and third-person perspectives are basic processes that are central to many higher-level social-cognitive processes (Decety & Lamm, 2006). For instance, in classical theory-of-mind tasks one needs to inhibit one’s *self* perspective in order to make inferences about what another person may know or see (Young, Dodell-Feder, & Saxe, 2010). When coordinating a joint action with another person, one needs to be able to share a common representation with the other person (Sebanz, Bekkering, & Knoblich, 2006), while at the same time keeping a representation of the actions that one needs to perform oneself (Wenke et al., 2011). Several studies have shown that people have an automatic tendency to take another person’s perspective (Samson, Apperly, Braithwaite, Andrews, & Scott, 2010; for a critical perspective on this issue, however, see Santiesteban, Catmur, Hopkins, Bird, & Heyes, 2014), and accordingly, social interaction requires an active process of switching between *self* and *other* perspectives (Brass, Ruby, & Spengler, 2009). The right temporoparietal junction (rTPJ) plays a key role in this process of switching between *self* and *other* perspectives (Santiesteban, Banissy, Catmur, & Bird, 2012; Sowden & Catmur, 2015). The rTPJ is a broadly defined region (Mars et al., 2012) that receives and integrates signals from different sensory modalities, such as visual, auditory, vestibular, tactile, and proprioceptive signals (Blanke, 2012). It has been argued that two key functions of the rTPJ are “social cognition” and “attention” (Scholz, Triantafyllou, Whitfield-Gabrieli, Brown, & Saxe, 2009).

Evidence for the role of the rTPJ in social cognition comes from studies on mentalizing and perspective taking. In classical mentalizing tasks (also called *theory-of-mind tasks*), participants are required to reflect on situations that require distinguishing between different perspectives (e.g., “Sally does not know that Ann knows that the ball is in the blue box”), and in these tasks the bilateral TPJ, the medial prefrontal cortex, and the superior temporal sulcus are consistently found to be activated (Gallagher & Frith, 2003; Van Overwalle & Baetens, 2009). Furthermore, patients with lesions to the left TPJ have shown impaired performance on theory-of-mind tasks (Samson, Apperly, Chiavarino, & Humphreys, 2004), and disrupting activity in the rTPJ through repetitive transcranial magnetic stimulation (TMS) results in impairments in different aspects of mentalizing tasks (Costa, Torriero, Oliveri, & Caltagirone, 2008; Young, Camprodon, Hauser, Pascual-Leone, & Saxe, 2010). Accordingly, it has been argued that the rTPJ is specifically involved in distinguishing between *self* and *other* perspectives in mentalizing tasks (Vogeley & Fink, 2003).

Other studies have used the own-body transformation task as a direct measure of spatial perspective taking. In this task, participants are required to adopt the spatial perspective of an avatar by imagining themselves in the perspective of the avatar (Arzy, Thut, Mohr, Michel, & Blanke, 2006; Blanke et al., 2005; Ganesh, van Schie, Cross, de Lange, & Wigboldus, 2015; van Elk & Blanke, 2014). In an alternative version of the own-body transformation task, participants are required to indicate the position of objects as seen from the perspective of another person (Kessler & Thomson, 2010; Vogeley et al., 2004). In fMRI studies using this task, stronger bilateral activation of the TPJ has been observed for adopting an egocentric as compared to an allocentric perspective (Ganesh et al., 2015; Zacks, Vettel, & Michelon, 2003), also suggesting a critical role of this region for distinguishing between *self* and *other* perspectives.

Interestingly, individual differences in performance on mental body transformation (MBT) tasks have been related to different personality and social-cognitive factors. For instance, researchers have found that schizotypal personality features are correlated to the duration of activity in the TPJ during an MBT task (Arzy, Mohr, Michel, & Blanke, 2007). In addition, men’s reaction times to mental rotations that implied a disembodied perspective increased with higher scores on a perceptual aberration scale (Mohr, Blanke, & Brugger, 2006). Participants who have reported an out-of-body experience (OBE) showed stronger switch costs for switching between the *self* and *other* perspectives in an MBT task (Easton, Blanke, & Mohr, 2009), but when OBE participants were presented with mental rotations implying a disembodied perspective, their performance on the MBT task was actually enhanced relative to controls (Braithwaite, Broglia, Bagshaw, & Wilkins, 2013). Furthermore, gender differences have been observed on MBT task performance, and for women MBT performance was related to their empathy scores (Mohr, Rowe, & Blanke, 2010). More recently, it was found that oxytocin administration makes men’s performance on MBT tasks more comparable to that of women (Theodoridou, Rowe, & Mohr, 2013). Together, these findings suggest that MBT tasks may actually tap into processes of both exocentric perspective taking and socio-cognitive functioning, in line with the proposed roles of the rTPJ in these domains.

Evidence for the causal role of the rTPJ in spatial perspective taking has been found in studies using noninvasive brain stimulation (Donaldson, Rinehart, & Enticott, 2015). Disruption of activity in the rTPJ through rTMS resulted in an impaired performance on the own-body transformation task, such that participants were slower in taking the perspective of another person (Blanke et al., 2005). Two recent studies using transcranial direct current stimulation (tDCS), suggest that stimulation of the rTPJ results in an enhanced ability to switch between the self and other perspective (Santiesteban et al., 2012; Santiesteban, Banissy, Catmur, & Bird, 2015). During tDCS a weak constant electric current is passed between an active and a reference electrode on the scalp. This causes enhanced (anodal) or decreased (cathodal) cortical excitability or no change in cortical excitability (sham condition; for a review, see Jacobson, Koslowsky, & Lavidor, 2012). It was found that anodal as compared to cathodal stimulation of the rTPJ resulted in a reduced interference effect in a control-of-imitation task (Santiesteban et al., 2012, 2015). These findings are further corroborated by a recent study, in which it was found that disruption of activity in the rTPJ through rTMS, resulted in an increased imitative compatibility effect (Sowden & Catmur, 2015), which was also interpreted as an impaired ability to suppress the other’s perspective.

In the present study, we built on and extended these findings in at least two ways. First, we aimed to investigate the contribution of the rTPJ to spatial perspective taking, while controlling for the effects of spatial attention. Previous studies had used a perspective-taking task that may have encouraged the use of a spatial strategy (i.e., if the avatar faces away from me respond with the same hand; if the avatar faces me, respond with the opposite hand; cf. ter Horst, Jongsma, Janssen, Van Lier, & Steenbergen, 2012; ter Horst, van Lier, & Steenbergen, 2010; van Elk & Blanke, 2014) rather than a perspective-taking strategy. Especially, an MBT task in which only front- and back-facing avatars are presented encourages the use of a contingency-based strategy rather than a process of exocentric perspective taking (Braithwaite & Dent, 2011). Similarly, the imitation task that was used in the studies by Santiesteban et al. (2012, 2015) and Sowden and Catmur (2015) also has a strong spatial compatibility component (Brass, Bekkering, Wohlschlager, & Prinz, 2000).^1^ Because the rTPJ is also involved in directing spatial attention (Corbetta & Shulman, 2002; Donaldson et al., 2015; Mars et al., 2012), reducing activity in the rTPJ should result in a reduced capacity to shift attention and stronger interference from irrelevant distractors—which matches exactly the pattern that was observed in these studies. Therefore, in our study we directly investigated the effects of tDCS on the rTPJ by comparing performance on two different tasks that were presented alternately in short mini-blocks. In the *MBT condition* (Blanke, Landis, Spinelli, & Seeck, 2004; Braithwaite, Samson, Apperly, Broglia, & Hulleman, 2011; Ganesh et al., 2015; Parsons, 1987; van Elk & Blanke, 2014), participants were required to indicate the laterality of a bracelet on the left or right arm as seen from the perspective of the avatar. In contrast, in the *control condition* participants were required to indicate whether the bracelet was on the left or the right side of the fixation cross, as seen from their own perspective.

**Fig. 1:**
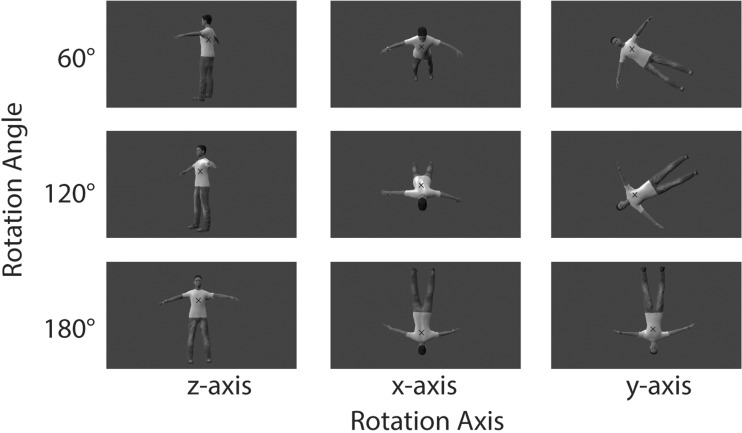
Stimuli used in the experimental task. Participants were presented with an on-screen avatar that was rotated along different rotation axes (*z*-, *x*-, and *y*-axes) and at different rotation angles (60°, 120°, –60°, –120°, 180°). Participants were instructed to indicate the position of the bracelet either with respect to the perspective of the avatar (i.e., mental body transformation task) or with respect to the fixation cross, as seen from their own perspective (i.e., control task).

Second, we aimed to increase the reliance on an embodied perspective-taking strategy in MBT tasks (Braithwaite & Dent, 2011; Kessler & Thomson, 2010), by including stimuli presenting an avatar along different orientations with respect to the participants. Previous studies have shown that in mental transformation tasks the reliance on a spatial strategy decreases and the engagement in a process of embodied spatial perspective taking increases when the stimuli are rotated along different rotational axes (Parsons, 1987; ter Horst et al., 2012; ter Horst et al., 2010). In a previous study, a novel variant of a mental body rotation task was introduced (labeled the “human own-body transformation” [HOBT] task), in which avatars were presented from perspectives above and below (Braithwaite et al., 2013). Participants who had reported having had an OBE-like experience showed improved performance on the HOBT task, suggesting that a task in which participants are required to adopt different spatial perspectives more strongly taps into the process of exocentric spatial perspective taking. In the present study, we used novel stimuli in which an avatar was presented in different orientations and was rotated along different axes (see Fig. 1)—comparable to the different axes and rotations that have been used in classical studies on the mental rotation of body parts (Parsons, 1987).

As we indicated above, some previous studies have reported bilateral TPJ activation in association with theory-of-mind reasoning and MBT tasks (Gallagher & Frith, 2003; Ganesh et al., 2015; Samson et al., 2004; Van Overwalle & Baetens, 2009; Zacks, 2008), whereas other studies have shown a specific role of the left TPJ in embodied spatial perspective taking (Blanke et al., 2005; Costa et al., 2008; Donaldson et al., 2015; Santiesteban et al., 2012, 2015). Although the specific functions of the left and right TPJ in spatial perspective taking are currently unknown (Donaldson et al., 2015), in the present study we decided to stimulate the right TPJ to allow for a direct comparison with previous tDCS studies (Hogeveen et al., 2015; Santiesteban et al., 2012, 2015). Following previous studies that have implicated the rTPJ in the control of online self–other representations (cf. Hogeveen et al., 2015; Santiesteban et al., 2012, 2015), we expected that anodal as compared to cathodal and sham tDCS would enhance performance on the MBT task, relative to the control condition. Specifically, an effect of activation of the rTPJ should become apparent on the MBT task through faster reaction times and reduced errors for anodal as compared to cathodal and sham stimulation, for stimuli implying a third-person perspective (i.e., front-facing stimuli/stimuli implying a large rotation angle from the participant’s own perspective), whereas no difference would be expected between conditions for stimuli implying a first-person perspective (i.e., back-facing stimuli/stimuli implying a short rotation angle from the participant’s own perspective). Previous studies have shown that vestibular stimulation (i.e., through a rotating chair) affects performance on an MBT task (van Elk & Blanke, 2014), and interestingly, in a recent study it was found that vestibular stimulation affected spatial perspective taking only during the first minute following stimulation (Gardner, Stent, Mohr, & Golding, 2016)—suggesting that experimentally induced changes in performance on MBT tasks may be especially apparent in the early phases of the task. In the present study, we investigated this in an explorative fashion, by comparing performance on the MBT task during the early and late phases of tDCS.

In addition to measuring the effects of tDCS on an MBT task, we included individual difference measures that have previously been associated with altered functioning of the rTPJ. More specifically, following Braithwaite et al. (2013; Braithwaite et al., 2011) we included the Launay–Slade hallucination scale (Braithwaite et al., 2011; Launay & Slade, 1981) as a measure of *aberrant perceptual beliefs*, including general hallucination proneness. The Cardiff Anomalous Perception Scale (Bell, Halligan, & Ellis, 2006; Braithwaite et al., 2013) was included as a measure of anomalous *perceptual experience*, and it also includes items related to temporal lobe instability and distorted body processing—which may be especially relevant for the present study. Finally, following Johnstone et al. (2012), we included the spiritual transcendence scale (Piedmont, 1999), which measures the propensity for self-transcendence in general. Given the involvement of the rTPJ in theory-of-mind tasks, we included a classical false-belief story task as a measure of theory-of-mind reasoning (Dodell-Feder, Koster-Hale, Bedny, & Saxe, 2011). These scales were included in exploratory analyses, to investigate whether the effects of tDCS on spatial perspective taking was further moderated by individual differences in hallucinatory experiences, distorted body processing, spiritual transcendence, and theory-of-mind reasoning.

Importantly, we explicitly note that the sample size that we used in our study was rather small—especially because quite a few participants dropped out from the final analysis. Therefore, the results should be interpreted with caution—especially in light of recent discussions on the efficacy of tDCS in general (Horvath, Forte, & Carter, 2015). However, we argue that publication of these findings is still important because (1) our findings potentially shed light on the boundary conditions of tDCS (online but not offline effects), (2) our findings appear to contradict previously published results regarding the causal role of the rTPJ in spatial perspective taking (Hogeveen et al., 2015; Santiesteban et al., 2012, 2015), and (3) to reduce the potential file-drawer problem in the field.

## Method

### Participants

In total, 58 men participated in the study. In this study we tested only male participants, to rule out the effects of hormonal fluctuations in women on the tDCS results (Inghilleri et al., 2004; Smith, Adams, Schmidt, Rubinow, & Wassermann, 2002). However, 11 participants dropped out because tDCS stopped too early during the experimental session (i.e., the impedance threshold was crossed during stimulation, and as a consequence stimulation stopped). Since the tDCS apparatus does not allow online monitoring of impedance, this effect was only observed at the end of the study, and therefore could not be avoided. In addition, two participants were excluded because they made errors on more than 20% of all trials on the MBT task or because of missing reaction time data in one of the cells. Thus, 45 participants were included in the final analysis (mean age = 21.16 years, *SD* = 2.61). The participants were randomly assigned to either the anodal (*N* = 16; mean age = 21.1 years), cathodal (*N* = 15; mean age = 21.4 years), or sham (*N* = 14; mean age = 21.0 years) condition. All participants gave their written informed consent before the start of the study. The procedures were approved by the Ethics Committee of the psychology department at the University of Amsterdam.

### Experimental setup and procedure

Participants were invited to the lab for two different sessions: a screening session of 1 h and an experimental session of 1.5 h. In the first screening session, the participants read the information brochure of the study, gave their informed consent, and completed five questionnaires to measure individual differences (see below). After completing the questionnaires, participants received tDCS for 60 s to experience its effects. Participants were offered the opportunity to withdraw from the study if they felt too uncomfortable with the procedure. One participant decided not to take part in the second session after this first tDCS.

In the experimental session, the participants started with 20 min of tDCS, which was applied with a neuroConn DC stimulator. Two 5 × 7 cm electrodes were attached to the scalp with Ten20 conductive electrode paste (Weaver, Colorado, USA). For the anodal condition, the anodal electrode was placed on CP6 and the reference electrode on C3, according to the 10–20 electroencephalographic system. The CP6 location roughly corresponds to the right supramarginal gyrus (BA40; cf. Koessler et al., 2009). The rationale for placing the control electrode at the C3 position (rather than at, e.g., Cz) was to avoid the potential problem that the current would flow through the skin and/or the cerebrospinal fluid, rather than through the brain (Donaldson et al., 2015). For the cathodal condition, the positions of the anodal and reference electrodes were reversed. Stimulation in the anodal and cathodal conditions was applied for 20 min with a 1-mA current. For the sham condition, electrodes were placed in the same locations as in the anodal condition, and stimulation was ramped up for 60 s, after which the stimulation was switched off. The DC stimulator was placed out of sight of the participants, to avoid participants inferring the condition that was being applied. Following previous studies using tDCS applied to the rTPJ (Hogeveen et al., 2015; Santiesteban et al., 2012, 2015), we used a *between-subjects* experimental design, in which participants were randomly assigned to either the anodal, cathodal, or sham condition. Although many tDCS studies have used a within-subjects design (e.g., Jacobson et al., 2012), in the present study we opted for a between-subjects design to avoid practice-related effects (Greenwald, 1976)—especially because the MBT task that was used in the present study is sensitive to participants developing a particular strategy for responding (Braithwaite et al., 2013; Kessler & Thomson, 2010).

During the 20-min block of anodal, cathodal, or sham tDCS, participants conducted an MBT task and a control task, which were presented alternately in short mini-blocks consisting of 40 trials each. The avatars were presented for a maximum duration of 10 s or until the participant responded. The interstimulus interval varied randomly between 2,000 and 3,000 ms. For the MBT task, we used stimuli representing a human-like avatar (see Fig. 1). The avatar was designed with MakeHuman (www.makehuman.org/), an open source tool for making 3-D characters, and Blender (https://www.blender.org/), an open source 3-D animation program. To create different stimuli, the avatar was rotated along three different rotation axes, the *z*-, *x*-, and *y*-axes (corresponding, respectively, to rotations along the yaw, pitch, and roll axes), and along six different rotation angles, 0°, 60°, 120°, –60°, –120°, and 180° (see Fig. 1). Note that the 0° condition was the same for all rotation axes and was not included in the statistical analysis of the data. In addition, in the main analysis we collapsed the stimuli oriented 60° and –60° and those oriented 120° and –120°, because these angles imply the same amounts of mental rotation. The rationale for including stimuli rotated along different axes and angles was that this manipulation increased the need to actually adopt the perspective of the avatar in the *other*-perspective-taking task (see below), rather than simply using an abstract rule for responding (e.g., “respond with the opposite hand if the avatar is facing me”; cf. ter Horst et al., 2010). In the MBT task, a gray fixation cross was overlaid on the picture; in the control task, a black fixation cross was displayed over the stimulus. Thus, the color of the fixation cross served as a reminder for the participant of which task they were expected to perform (see below).

In the *MBT task*, participants were instructed to indicate whether a bracelet was on the right or the left arm *as seen from the perspective of the avatar on the screen*. In the *control task*, participants had to indicate whether the bracelet was on the left or the right side of the cross on the torso of the avatar with respect to the central fixation cross on the screen, *as seen from their own perspective*. Each task (i.e., MBT vs. control) was presented in five mini-blocks consisting of 40 trials each, and the blocks were presented in a randomized order. Thus, in total, each task consisted of 200 trials. Participants were required to respond within 10 s, and after they had responded the next stimulus appeared. Participants responded by pressing the left or right arrow keys on a keyboard with their right hand. The experiment was programmed with the Presentation software (NeuroBehavioral Systems, Berkeley, USA).

At the beginning of the experiment, participants performed eight practice trials for each experimental task. The specific instructions presented to the participants prior to the study were as follows:

In this study you will be required to respond to pictures of an avatar, which is placed in different positions. During the experiment you will be required to perform two alternating tasks. In the first task (when the fixation cross on the picture is gray) you are required to indicate whether a red bracelet is on the left or the right wrist of the avatar—as seen from the position of the avatar. You should thus try to mentally take the perspective of the avatar. Although there are several possible ways to adopt the avatar’s perspective, in this study we are particularly interested in mental rotation of your body. Thus, we ask you to mentally rotate your body in the position of the avatar during the experiment. If the bracelet is on the left wrist of the avatar, you can respond by pressing the left button. If the bracelet is on the right wrist of the avatar, you can respond by pressing the right button. In the second task you will be presented with a similar avatar—however the avatar will have a black cross on his torso. In this task you are required to indicate whether the red bracelet is left or right with respect to the cross—as seen from your own perspective. In this task you should thus adopt your own perspective. If the bracelet is on the left side with respect to the cross, you can respond by pressing the left button. If the bracelet is on the right side with respect to the cross, you can respond by pressing the right button. Please try to respond as fast and accurately as possible.

When the instructions were clear, the tDCS was started and participants started the experiment. Participants were allowed to take short, self-paced breaks between blocks and were instructed to continue with the next block by pressing the Enter button on the computer keyboard. We note that on average completing the experimental task took longer (mean = 25 min, *SD* = 4.2, range = 19.4–42.8 min) than the duration of the tDCS.

Participants also participated in a full-body illusion (FBI) experiment (Ehrsson, 2007), following the tDCS and the experimental task. A virtual reality setup was used in which participants viewed themselves as filmed on the back through a head-mounted display. In different blocks, the experimenter applied either synchronous or asynchronous visuotactile stimulation to the participant’s chest. The effects of the induction of the full-body illusion were measured using both questionnaire data, and the crossmodal congruency task was used to measure the effects of the FBI on multisensory integration (Aspell, Lenggenhager, & Blanke, 2009). We decided not to report these data in the present article, but we note that tDCS did not affect the FBI, as measured by the self-report measures and the crossmodal congruency task.

### Individual difference measures

Different measures were included to assess individual differences in the relevant personality traits. We used the STS (cf. Piedmont, 2001), which measures individual differences in spirituality. The STS consists of 24 items concerning spiritual experiences (e.g., “I feel on a higher level all of us share a common bond”; “I find inner strength and/or peace from my prayers or meditations”) with a 7-point scale ranging from *I totally disagree* to *I totally agree*. The STS results were normally distributed, with a mean of 3.32 (*SD* = 0.93, range = 1.67–5.67), *p* = .34 (Shapiro–Wilk), Cronbach’s *α* = .90.

The LSHS (Braithwaite et al., 2011; Launay & Slade, 1981) was used to measure hallucinatory experiences that people may have experienced in the past. The LSHS consists of 12 items concerning different types of hallucinations (e.g., “Sometimes my thoughts seem as real as actual events in my life”; “I often hear a voice speaking my thoughts aloud”) with a 7-point scale ranging from *I totally disagree* to *I totally agree*. The LSHS results were also normally distributed, with a mean of 2.71 (*SD* = 0.68, range = 1.33–4.33), *p* = .32 (Shapiro–Wilk), Cronbach’s *α* = .78.

We used the CAPS (cf. Bell et al., 2006; Braithwaite et al., 2013), consisting of 31 yes/no items to measure perceptual anomalies (e.g., “Do you ever sense the presence of another being, despite being unable to see any evidence?” or “Do you ever see shapes, lights, or colors even though there is nothing really there?”). Participants were required to indicate whether they were familiar with the experience described by selecting a “yes” or a “no” response, and the total number of “yes” responses was calculated per participant. The CAPS results were not normally distributed, *p* = .0024 (Shapiro–Wilk), Cronbach’s *α* = .78, and therefore it was not included as a covariate in the analysis.

Finally, we used a false-belief story (FBS) task (Dodell-Feder et al., 2011), consisting of 20 memory control items and 20 items in which participants were required to make inferences about the mental states of others. An example of a mental false-belief item is: “The morning of high school dance Sarah placed her high heel shoes under her dress and then went shopping. That afternoon, her sister borrowed the shoes and later put them under Sarah’s bed. *Sarah gets ready assuming her shoes are under the dress.*” An example of a false-belief physical item is “When Jeff got ready this morning he put on a light pink shirt instead of a white one. Jeff is color-blind, so he can’t tell the difference between subtle shades of color. *In reality, Jeff’s shirt is pink*.” For the FBS task, we calculated the total number of correct responses to the mental items per participant. However, due to performance being close to ceiling level, we decided not to report these data in the present article (we note, however, that no effect of tDCS on performance was apparent in the FBS task).

### Data analysis

Our main analysis focused on the effects of our tDCS manipulation (anodal, cathodal, sham) on our dependent measures. Incorrect responses and reaction times that exceeded that participant’s mean by more than two standard deviations were excluded from the reaction time analysis. As we indicated above, completing the experimental task took longer than the tDCS, and as a consequence, the reaction time data during the final part of the experimental task were collected when no online tDCS was being applied. In addition, over time participants became more efficient in conducting the experimental task, as indicated by subjective reports at the end of the experiment and the overall declines in reaction times and error rates over the course of the experimental task (see below). Accordingly, it could well be that the effects of tDCS on our experimental task were most pronounced during the first than during the second half of the experiment. Indeed, previous studies using tDCS have shown that online stimulation can affect performance already within the first 5 min following stimulation (Fecteau et al., 2007; Filmer, Dux, & Mattingley, 2014; Wirth et al., 2011). In addition, a recent study revealed that vestibular stimulation already affected performance on an MBT task within a minute after stimulation (Gardner et al., 2016). Thus, to investigate the possibility of early vs. late effects of tDCS on MBT task performance, we included Block (first vs. second half of the experiment) as an additional within-subjects factor in our analysis.^2^ We thus performed an analysis of the reaction times and the error rates using a repeated measures analysis of variance (ANOVA) with Condition (anodal vs. cathodal vs. sham) as a between-subjects factor and Block (first half, second half), Rotation Axis (*z*-axis, *x*-axis, *y*-axis), and Rotation Angle (60°, 120°, 180°), as well as the two tasks (MBT vs. control task), as within-subjects factors. For exploratory purposes, we also looked at the role of individual differences in our tDCS manipulation. Toward this end, we included the different scales (LSHS, STS) as covariates in our repeated measures ANOVA.

## Results

### Reaction times

On average, participants made errors on 3.10% of all trials in the experimental task, and 4.37% of all trials were removed because the reaction times exceeded the participant’s mean by more than two standard deviations.

**Fig. 2:**
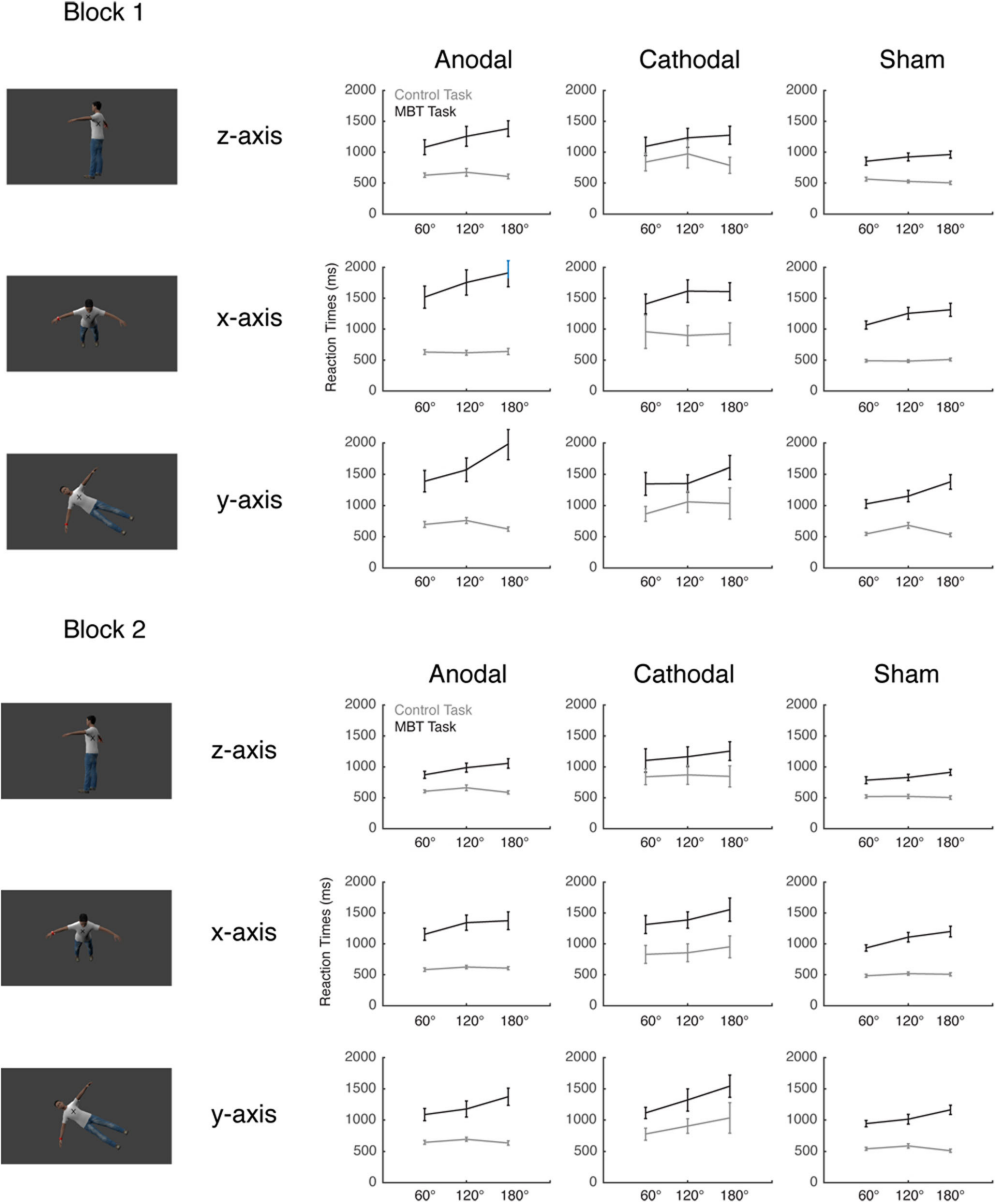
Reaction times during the first half of the experimental task (upper panel) and the second half of the experimental task (lower panel), as a function of rotation angle and task (gray lines = control task; black lines = mental body transformation [MBT] task). Graphs on the left represent the anodal tDCS condition, middle graphs the cathodal tDCS condition, and right graphs the sham condition. The upper row represents reaction times to stimuli rotated along the *z*-axis, the middle row those to stimuli rotated along the *x*-axis, and the lower row stimuli rotated along the *y*-axis. Error bars represent standard errors.

The outcomes from the ANOVA including Block as a within-subjects factor are reported in Table 1, and the data are presented in Fig. 2. As expected, a main effect of block, *F*(1, 41) = 24.42, *p* < .001, *η* ^2^ = .37, reflected that participants responded faster in the second (mean RT = 825 ms, *SE* = 40) than in the first (mean RT = 946 ms, *SE* = 56) half of the experiment. In addition, a main effect of task was observed, *F*(1, 43) = 93.6, *p* < .001, *η* ^2^ = .70, reflecting that participants responded more slowly during the MBT task (mean RT = 1,179 ms, *SE* = 65) than during the control task (mean RT = 616 ms, *SE* = 32).

**Table 1:** Analysis of variance for the reaction time data from the MBT task

Effect	*df*	*F*	*MSE*	*p*	*η* ^2^
Block	1,41	24.42	4,445,002.0	.000	.37
Task	1,41	93.60	125,704,133	.000	.70
Axis	2,82	89.86	4,323,736.9	.000	.69
Angle	2,82	56.46	2,280,597.1	.000	.58
Block × Task	1, 41	16.00	2,086,636.5	.000	.28
Block × Task × Condition	2, 41	6.00	781,030.6	.005	.23
Block × Axis	2, 82	8.31	95,376.4	.001	.17
Task × Axis	2, 82	72.04	3,811,376.1	.000	.64
Block × Task × Axis	2, 82	5.94	56,427.9	.004	.13
Task × Angle	2, 82	69.55	2,622,869.2	.000	.63
Block × Task × Angle	2, 82	3.56	44,266.0	.033	.08
Block × Task × Angle × Condition	4, 82	2.80	34,821.8	.031	.12
Axis × Angle	4, 164	3.65	76,335.0	.007	.08
Task × Axis × Angle	4, 164	9.28	175,064.4	.000	.19

A main effect of rotation axis, *F*(2, 82) = 89.86, *p* < .001, *η* ^2^ = .69, reflected that participants were faster in rotating along the *z*-axis (mean RT = 793 ms, *SE* = 36) than along either the *x*-axis (mean RT = 953 ms, *SE* = 46, *p* < .001) or the *y*-axis (mean RT = 947 ms, *SE* = 47, *p* < .001).

As expected, a main effect of rotation angle, *F*(2, 82) = 56.46, *p* < .001, *η* ^2^ = .58, reflected that reaction times increased with larger rotation angles: Participants responded faster to 60° stimuli (mean RT = 827 ms, *SE* = 39) than to 120° stimuli (mean RT = 911 ms, *SE* = 44, *p* < .001) or 180° stimuli (mean RT = 957 ms, *p* < .001), and they responded faster to 120° stimuli than to 180° stimuli (*p* = .003).

These main effects were qualified by significant two- and three-way interaction effects (see Table 1). The three-way interaction reflects that the mental rotation effects (slower reaction times with increased rotation angles) differed as a function of the task and the rotation axis (see Fig. 2), thereby replicating previous findings using similar stimuli (Ganesh et al., 2015; Parsons, 1987).

Importantly, next to the expected significant main effects and interaction effects between task, axis, and rotation angle, we also found signification interactions between block, condition, and task, *F*(2, 41) = 6.00, *p* = .005, *η* ^2^ = .23, and between block, condition, task, and rotation angle, *F*(4, 82) = 2.80, *p* = .031, *η* ^2^ = .12 (see Table 1). To explore the source of these interaction effects, we conducted separate analyses for Block 1 and for Block 2, which confirmed that the effects of condition were completely driven by Block 1 (i.e., in Block 2, condition did not interact with any of the other factors; *F* < 1, n.s.).

The reaction time data for Block 1 are presented at the top of Fig. 2. Here we report only significant interaction effects with Condition as an experimental factor—since these are our main effects of interest (for a full report of all significant main effects and interactions, see Table 2). We found significant interactions between condition and angle, *F*(4, 84) = 3.12, *p* = .019, *η* ^2^ = .12; between condition, task, and axis, *F*(4, 84) = 2.75, *p* = .033, *η* ^2^ = .12; and between condition, task, and angle, *F*(4, 84) = 4.12, *p* = .004, *η* ^2^ = .16.

**Table 2:** Analysis of variance for the data from the first half of the experiment (during tDCS)

Effect	*df*	*F*	*MSE*	*p*	*η* ^2^
Task	1, 42	77.75	88,224,938.0	.000	.65
Axis	2, 84	81.07	3,176,624.1	.000	.66
Angle	2, 84	54.24	1,603,850.1	.000	.56
Task × Axis	2, 84	72.94	2,601,276.6	.000	.635
Task × Angle	2, 84	66.70	1,861,884.9	.000	.614
Axis × Angle	4, 168	2.95	81,281.7	.022	.066
Task × Axis × Angle	4, 168	7.35	163,777.8	.000	.15
Task × Condition	2, 42	3.05	3,457,915.9	.058	.13
Axis × Condition	4, 84	2.46	96,179.22	.052	.11
Angle × Condition	4, 84	3.12	92,320.6	.019	.13
Task × Axis × Condition	4, 84	2.75	98,019.1	.033	.12
Task × Angle × Condition	4, 84	4.12	114,964.6	.004	.16

As can be seen in Fig. 2, these interaction effects seemed to be driven primarily by slower reaction times for the anodal condition in the MBT task—specifically for stimuli with high rotation angles, and most pronounced for stimuli rotated along the *x*- and *y*-axes. To explore the source of the interaction effects, separate post-hoc tests were conducted for the MBT task and the control task. These post-hoc analyses confirmed that the tDCS did not affect reaction times for the control task, *F* < 1.65, *p* = .169, *η* ^2^ = .07. For the MBT task, interactions were found between condition and rotation axis, *F*(4, 84) = 2.70, *p* = .036, *η* ^2^ = .11, and between condition and rotation angle, *F*(4, 84) = 4.10, *p* = .004, *η* ^2^ = .16.

To further investigate these interaction effects, additional post-hoc analyses were conducted by collapsing the data across all rotation axes or all rotations angles, respectively. Given our initial hypotheses, we predicted a specific effect of anodal stimulation relative to both cathodal and sham stimulation, and thus a Helmert contrast was used. With respect to *rotation angle*, participants in the anodal condition responded marginally slower than those in the cathodal and sham conditions for 60° stimuli (mean difference = 280, *SE* = 153; *p* = .074, 95% CI = [–29 589]) and 120° stimuli (mean difference = 354, *SE* = 186; *p* = .064, 95% CI = [–21 728]), and significantly slower for 180° stimuli (mean difference = 505, *SE* = 197; *p* = .014, 95% CI = [107 903]).

With respect to *rotation axis*, participants in the anodal condition responded slower than in the cathodal and sham conditions for stimuli rotated along the *x*-axis (mean difference = 447, *SE* = 197; *p* = .028, 95% CI = [51 844]) and the *y*-axis (mean difference = 432, *SE* = 203; *p* = .039, 95% CI = [23 841]), and marginally slower for stimuli rotated along the *z*-axis (mean difference = 258, *SE* = 135; *p* = .062, 95% CI = [–13.5 530]). Condition did not interact with the error rates (*F* < 1). We note that these post-hoc tests were not corrected for multiple comparisons, and therefore these effects must be interpreted with caution.

### Moderation analyses

To investigate the possibility that individual differences moderated the effects of tDCS on the experimental task, in a subsequent analysis we included the questionnaire data as an additional factor in our analysis, while looking specifically at the between-subjects interaction term between condition and the individual difference measures (LSHS and STS). The LSHS and STS results were not significantly correlated, *p* > .1. Two separate ANOVAs were conducted while including one scale at a time as a covariate. We note that the moderation analyses were conducted only on the data from the first experimental block, because effects of tDCS were only apparent in the first part of the study. To facilitate interpretation of the moderation effects, for each scale a median split was conducted, creating groups of participants who scored low versus high on each measure (see Table 3). However, in all analyses the individual difference score was treated as a continuous variable.

**Table 3:** Reaction times for the mental body transformation task as a function of condition (anodal, cathodal, or sham stimulation) and rotation angle (60°, 120°, 180°), and according to whether participants scored below (left side of table) or above (right side of table) the median on the Launay–Slade hallucination scale (LSHS) and the spiritual transcendence scale (STS)

	Rotation Angle			Rotation Angle		
Condition	60°	120°	180°	60°	120°	180°
	LSHS Low			LSHS High		
Anodal	**1,428 (165)**	**1,650 (200)**	**1,963 (211)**	1,209 (187)	1,397 (227)	1,586 (239)
Cathodal	994 (187)	1,087 (227)	1,237 (239)	1,199 (175)	1,377 (212)	1,453 (224)
Sham	1,110 (175)	1,262 (212)	1,389 (224)	855 (202)	955 (245)	1,027 (259)
	STS Low			STS High		
Anodal	**1,306 (174)**	**1,536 (209)**	**1,829 (225)**	1,365 (198)	1,544 (237)	1,757 (255)
Cathodal	1,103 (185)	1,300 (221)	1,354 (239)	1,104 (192)	1,176 (233)	1,351 (248)
Sham	938 (208)	1,061 (251)	1,170 (268)	1,047 (180)	1,183 (218)	1,282 (232)

Standard errors are presented between parentheses. The most relevant findings that are referred to in the Results section are in bold.

First, we established that the participants in the different groups did not differ in their scores on the individual difference measures (*F* < 1.9, *p* > .158). For the *LSHS*, a significant interaction was found between condition, LSHS, task, and rotation angle, *F*(6, 82) = 3.33, *p* = .006, *η* ^2^ = .20. A marginally significant interaction was found between condition, LSHS, task, and rotation axis, *F*(6, 82) = 2.05, *p* = .068, *η* ^2^ = .13. Post-hoc tests showed that the effects were driven by differences in the MBT task relative to the control task: For the MBT task, a significant interaction emerged between condition, LSHS, and rotation angle, *F*(6, 82) = 2.80, *p* = .016, *η* ^2^ = .17. This interaction reflected that the effect of anodal tDCS on rotation angle was most pronounced for participants scoring low on the LSHS (see Table 3). For the control task, no significant interactions between condition and the LSHS were observed (*F* < 1.65, *p* = .083).

For the *STS*, significant interactions were found between condition, STS, task, and rotation axis, *F*(6, 82) = 2.34, *p* = .039, *η* ^2^ = .15, and between condition, STS, task, and rotation angle, *F*(6, 82) = 2.75, *p* = .017, *η* ^2^ = .17. Post-hoc tests showed that for the MBT task, a significant interaction emerged between condition, STS, and rotation angle, *F*(6, 82) = 2.45, *p* = .032, *η* ^2^ = .15. This interaction reflected that the effect of anodal tDCS on rotation angle was most pronounced for participants scoring low on the STS (see Table 3). The interaction between condition, STS, and rotation axis was not significant, for either the MBT or the control task (*F* < 1.75, *p* = .12).

### Control for directionality effects

A main finding in the present study is that tDCS impaired third-person perspective taking, as reflected in slower reaction times in the MBT task for anodal than for cathodal and sham stimulation. In the present study, the cathodal electrode was positioned at the location corresponding to C3, which is located directly above the left premotor cortex (PMC). Accordingly, it could well be that the impairment observed in the anodal condition was actually related to a cathodal effect at C3 rather than an anodal effect at the CP6 location, because previous studies have shown that the premotor cortex is also involved in mental imagery (Lamm, Windischberger, Moser, & Bauer, 2007; Vingerhoets, de Lange, Vandemaele, Deblaere, & Achten, 2002; Zacks, 2008). Previous studies using a similar MBT tasks indicated that participants typically tend to imagine rotating their bodies along the shortest path, thereby implying imagined body transformations in a specific direction (i.e., clockwise [CW] or counterclockwise [CCW]; Ganesh et al., 2015; Parsons, 1987; van Elk & Blanke, 2014). In addition, studies on motor imagery have shown that activity in the left and in the right premotor cortex differs as a function of the directionality of the implied mental rotation (de Lange, Helmich, & Toni, 2006; ter Horst et al., 2012; Vingerhoets et al., 2002). On the basis of these finding, we could hypothesize that an eventual effect of cathodal stimulation at C3 should be reflected in a direction-specific impairment for CW as compared to CCW rotations.

To investigate this possibility, in an additional analysis we classified the stimuli according to their implied rotation directions (i.e., CW vs. CCW; note that this could only be done for stimuli rotated 60° and 120° and for stimuli rotated along the *z*-axis and the *y*-axis). The reaction time data were analyzed using a repeated measures ANOVA with the factors Task (MBT task vs. control task), Axis (*z*-axis vs. *y*-axis), Rotation Angle (60° vs. 120°), and Rotation Direction (CW vs. CCW) as within-subjects factors, and Condition (anodal vs. cathodal vs. sham) as a between-subjects factor. This additional analysis was conducted only on the data from the first block (similar to the moderation analyses reported above). No main effect of rotation direction was found, *F*(1, 42) < 2.73, *p* = .11, and rotation direction did not significantly interact with either task or condition, *F*(2, 42) = 2.02, *p* = .15. These findings indicate that the tDCS effects observed were likely not related to a direction-specific effect as a consequence of stimulation of the left premotor cortex.

## Discussion

The aim of the present study was to elucidate the functional role of the rTPJ in third-person perspective taking, as measured by using an MBT task. On the basis of the existing literature, we hypothesized that anodal as compared to cathodal and sham tDCS would enhance performance on the MBT task (i.e., as reflected in faster reaction times for stimuli implying a stronger body rotation) relative to the control condition. The main finding of this study was that anodal as compared to cathodal and sham stimulation of the rTPJ actually impaired performance on an MBT task as compared to the control task, reflected in slower reaction times on the MBT task. This effect was most pronounced for stimuli with an increased rotation angle with respect to the perspective of the participant, and for stimuli implying rotation along more difficult rotation axes (i.e., along the roll and pitch axes rather than the yaw axis). These findings suggest that—contrary to our initial predictions—stimulation of the rTPJ impairs third-person perspective taking, but it has no effect on first-person perspective taking (i.e., we did not observe an effect of tDCS in our control task).

### tDCS of the rTPJ impairs spatial perspective taking

Our findings are in apparent conflict with previous studies that have implicated the rTPJ in enhanced spatial perspective taking (Blanke et al., 2005; Santiesteban et al., 2012) and in supporting the online control of first- versus third-person perspective (Hogeveen et al., 2015; Santiesteban et al., 2012, 2015; Sowden & Catmur, 2015). This discrepancy may be related to differences in the tasks that were used, the stimulation sites, and cathodal effects on the contralateral hemisphere. First, the tasks that were used in previous studies on the causal role of the rTPJ in social cognition (Blanke et al., 2005; Hogeveen et al., 2015; Santiesteban et al., 2012; Sowden & Catmur, 2015) were strongly subject to strategy- and attention-related effects. Accordingly, it is unclear whether the effects of rTPJ stimulation in these studies were related to spatial perspective taking or to attentional processes, which are also mediated by the rTPJ (Corbetta & Shulman, 2002). In our study, no effect of tDCS on the control task was observed, thereby showing a selective effect of rTPJ stimulation on the MBT task, while controlling for effects on spatial attention (see also Sowden & Catmur, 2015). In addition to differences in the experimental tasks, also the position of the cathodal electrode in our study differed from those in previous studies (Santiesteban et al., 2012, 2015), since we positioned it above C3 instead of Cz. As a consequence of the electrode placement, it could be that the effects that we observed in the anodal condition were actually related to cathodal stimulation at the contralateral hemisphere (i.e., above the left motor cortex). We did not observe an effect of tDCS on the control task, thereby ruling out the potential confound that the effects observed were a mere consequence of overall slower reaction times due to inhibition of the motor system. However, because the premotor and somatosensory cortices have also been implicated in mental rotation and motor imagery (Lamm et al., 2007; Vingerhoets et al., 2002; Zacks, 2008), it could be that the effect of anodal stimulation on the MBT task was actually related to cathodal effects on motor- and somatosensory brain regions. Our additional analyses regarding a direction-specific effect on spatial perspective taking did not support this account, and in addition, no effect of cathodal rTPJ stimulation (and, consequently, of anodal C3 stimulation) was observed, thereby making this alternative explanation less likely.

Previous studies have indicated that the MBT tasks used in the present study strongly rely on exocentric (as compared to egocentric) visual processing, and that participants who experienced an out-of-body experience actually performed better on this task than did a control group (Braithwaite & Dent, 2011; Braithwaite et al., 2013; Braithwaite et al., 2011). These findings have been related to the disintegration of egocentric processing in OBE participants (a similar process could account for the increased switch costs observed in OBE participants in the MBT task; see Easton et al., 2009). Accordingly, our findings could suggest that activation of the rTPJ impairs third-person perspective taking through a process of increasing the reliance on a first-person perspective. This interpretation is in line with studies that have implicated the rTPJ in supporting an egocentric frame of reference (Maguire et al., 1998; Vogeley & Fink, 2003) and in maintaining a first-person perspective during MBT tasks (Ganesh et al., 2015; Zacks et al., 2003). In addition, previous studies have shown that activation of the rTPJ results in reduced interference from observed movements in a control-of-imitation task (Santiesteban et al., 2012), whereas deactivation of the rTPJ has been associated with increased imitative interference (Sowden & Catmur, 2015). These findings are also compatible with the view that stimulation of the rTPJ may facilitate a first-person as compared to a third-person perspective (Maguire et al., 1998; Vogeley & Fink, 2003). We can only speculate about the potential implications of these findings, but because previous studies have related performance on MBT tasks to empathy and other social-cognitive processes (Arzy et al., 2007; Braithwaite et al., 2011; Mohr et al., 2006; Mohr et al., 2010; Mohr, Rowe, Kurokawa, Dendy, & Theodoridou, 2013; Theodoridou et al., 2013), it could well be that activation of the rTPJ through tDCS facilitates egocentric processing, while actually impairing or reducing empathic perspective taking.

Throughout this article we have interpreted slower responses on the MBT task as reflecting a process of impaired third-person perspective taking. Thus, faster responses on the MBT task were taken to reflect more efficient, and slower responses to reflect more difficulty with, third-person perspective taking. This interpretation receives support from the finding of a classical linear increase of reaction times with both rotation angle and rotation axis, implying a more difficult or complex process of MBT (Ganesh et al., 2015; Parsons, 1987). In addition, previous studies have shown that vestibular stimulation facilitates reaction times on a mental perspective-taking task in a direction-specific fashion (Deroualle, Borel, Devèze, & Lopez, 2015; van Elk & Blanke, 2014). On the other hand, it could also be argued that slower reaction times in an MBT task actually reflect a stronger or deeper engagement in third-person perspective taking (e.g., adopting an “embodied” strategy for the MBT task, in which truly imagining rotating one’s body probably takes more time than a “disembodied” or rule-based strategy). This interpretation would be in line with several apparently contradictory findings, such as the observation that increased empathy is associated with slower responses on MBT tasks (Mohr et al., 2010; Theodoridou et al., 2013) and that perceptual aberrations are positively related to reaction times, potentially reflecting more effort in adopting a third-person perspective (Mohr et al., 2006). We suggest that a crucial factor determining the interpretation of reaction times as reflecting either facilitated or impaired perspective taking likely depends on the strategy employed by the participant to perform the task. In our study, we used stimuli that encouraged the need to rely on own-body transformations, and we explicitly instructed participants to use an embodied perspective-taking strategy. Therefore, we argue that our reaction time findings likely reflect that for participants in the anodal condition it became more difficult to engage in this process of “embodied third-person perspective taking”—although in future studies it will be important to include questions regarding the actual strategies used by participants.

Interestingly, individual differences in hallucination proneness and spiritual transcendence interacted with the effects of tDCS in our experimental task: The effect of anodal tDCS on the MBT task was most pronounced for participants who scored low on the LSHS and on the STS. The LSHS has previously been associated with hallucinatory visual and auditory experiences, and higher scores on this scale have been associated with having had an OBE (Braithwaite et al., 2011). Other studies have suggested that increased spiritual transcendence is associated with altered functioning of the right inferior parietal lobe and the rTPJ (Johnstone et al., 2012; Urgesi, Aglioti, Skrap, & Fabbro, 2010). Furthermore, it has been found that participants who have an OBE and who score high on measures of schizotypal perceptual aberrations show impaired performance on own-body transformation tasks (Blanke et al., 2005; Braithwaite et al., 2011; Mohr et al., 2006). Following the predictive-processing account of schizotypy, according to which visual and auditory hallucinations are primarily related to a disturbed error-monitoring process (Fletcher & Frith, 2009; Frith, 2005), difficulties on the MBT task may be caused by problems with using forward models to solve mental-imagery tasks (Wolpert & Kawato, 1998). According to this account, the impairment that we observed of anodal tDCS among participants scoring low on the LSHS and the STS may be related to impairment with using a forward model of one’s own body, thereby making their performance more similar to those who score high on these scales. We note that the effects of individual differences need to be interpreted with caution, given the relatively small sample size in our study, but still the suggestion that hallucination proneness and spirituality could function as important moderators of the effects of tDCS provides interesting avenues for future studies.

### Limitations

The effects of tDCS on spatial perspective taking were only observed during the first half of the experiment. This may have been related to two different factors that are difficult to disentangle within the present experimental design. First, many studies have shown that the MBT task that was used in the present study is prone to strategy-related effects, whereby participants infer a particular rule for responding (Kosslyn, Ganis, & Thompson, 2001; Murray, 1997; Tomasino & Rumiati, 2004), rather than engaging in a process of embodied spatial perspective taking (Kessler & Thomson, 2010). In the instructions we placed specific emphasis on the importance of using a strategy to mentally put oneself in the shoes of the avatar. In addition, by using stimuli rotated along different rotation axes, we aimed to increase the need to actually engage in a process of own-body transformation (Parsons, 1987; ter Horst et al., 2010). Still, given the length of the experiment, it is likely that with increased practice participants became more efficient in conducting the MBT task, and eventually developed an alternative strategy for responding—thereby reducing the potential impact of tDCS of the rTPJ over the course of the experiment.

In addition to this practice-related effect, the first half of the experimental task was conducted while online tDCS was applied, whereas the last part of the task was mainly conducted while tDCS was already switched off. tDCS is supposed to affect brain activity through two general mechanisms (Stagg & Nitsche, 2011). The first mechanism proposes that tDCS results in a short-term (lasting less than 5 min) shift in the resting membrane potential, such that the neurons proximal to the anodal electrode become hypopolarized, whereas neurons close to the cathodal electrode become hyperpolarized. However, the direction of the polarization also depends on the orientation of the neural tissue, and as a consequence the effects of tDCS may differ depending on which brain region is stimulated (Datta et al., 2009). The second mechanism proposes that tDCS results in a process similar to long-term potentiation under the anodal electrode, and long-term depression under the cathodal electrode, and this process is assumed to be active for an extended period of time (up to 120 min) following tDCS. The existing evidence is ambiguous with respect to the effects of short-term versus long-term tDCS (for general criticism of the efficacy of tDCS, see Horvath et al., 2015), with some studies reporting effects during online tDCS but no long-term effects (Wirth et al., 2011), but other studies reporting mixed evidence for online tDCS effects (Nitsche et al., 2005). Our findings support an online effect of tDCS of the rTPJ on spatial perspective taking. In contrast, two previous studies have shown long-term effects of tDCS of the rTPJ on a control-of-imitation task (Hogeveen et al., 2015; Santiesteban et al., 2012), but these studies differed in terms of the task that was used and did not directly compare online versus offline tDCS effects.

Alternatively, it could be that the effects observed in our study are related to preexisting differences between the groups in performance on the MBT task. Although all participants were given the same amount of practice, we did not include a baseline measure of MBT task performance. Accordingly, it could be that—due to individual variability—the anodal group at the start of our study (i.e., during the first half of the experimental block) already performed slower on the MBT task than did the cathodal and sham groups. According to this account, the absence of an effect of tDCS during the second half of the experiment could be related to the anodal stimulation actually facilitating OBT task performance—thereby making the reaction time pattern for the anodal group more comparable to those of the other groups. This account would be compatible with the directionality of tDCS effects observed in previous studies (Hogeveen et al., 2015; Santiesteban et al., 2012, 2015). We note, however, that the error data did not support the notion of an overall difference in task performance between the groups, and also that the reaction time pattern did not match a stronger difference in performance at the very beginning of the experimental task.^3^

### Recommendations for future studies

Future studies should further elucidate the boundary conditions for tDCS to have an effect on spatial perspective taking, by directly comparing performance during online versus offline tDCS. By including different and alternating tasks to measure spatial perspective taking (e.g., including different types of avatars and spatial perspectives), the potential confound of practice-related effects overshadowing the eventual effects of tDCS could be circumvented. Including a baseline measure on an MBT task would be important to allow for assessing potential preexisting differences in task performance between the groups. Future studies will also be required to settle the question of the extent to which the placement of the cathodal electrode affects the effects of TPJ stimulation. For studies targeting the TPJ, we recommend placing the cathodal electrode at the Cz location, to avoid possible interference from modulating lateralized motor responses. We also recommend tDCS protocols that allow experimenters to monitor the impedance of the tDCS electrodes online (i.e., rather than only at the end of the task), and we recommend using sufficiently powered sample sizes to allow for stronger claims regarding the absence or presence of a potential effect of tDCS.

## Conclusions

This study has shown that online stimulation of the rTPJ through anodal as compared to cathodal and sham tDCS results in an impaired ability to take a third-person perspective, as reflected in slower responses on an MBT task.

## Footnotes

1 We note however that the authors aimed to control for the effects of spatial compatibility in the imitation task by rotating the stimulus hand by 90 deg along the horizontal plane (Santiesteban et al., 2012, 2015; Sowden & Catmur, 2015). In addition, effects of tDCS were observed on a different perspective-taking task that was less susceptible to spatial compatibility effects.

2 Because reaction time data were missing for one participant for the 180° stimuli in the second block of the MBT task, the subsequent analysis was conducted using the data from 44 participants. The analyses on the data from the first block only were conducted with all 45 participants.

3 Instead, it seemed as if the anodal group specifically started to respond more slowly in the MBT task in the second half of the first block, in line with the suggestion that the impaired performance observed in this group was related to the tDCS.
